# RETRACTION: Novel C‐Terminal Heat Shock Protein 90 Inhibitors Target Breast Cancer Stem Cells and Block Migration, Self‐Renewal, and Epithelial–Mesenchymal Transition

**DOI:** 10.1002/1878-0261.70300

**Published:** 2026-07-09

## Abstract

**RETRACTION**: C. Subramanian, P. T. Grogan, T. Wang, J. Bazzill, A. Zuo, P. T. White, A. Kalidindi, D. Kuszynski, G. Wang, B. S. J. Blagg and M. S. Cohen, “Novel C‐Terminal Heat Shock Protein 90 Inhibitors Target Breast Cancer Stem Cells and Block Migration, Self‐Renewal, and Epithelial–Mesenchymal Transition,” *Molecular Oncology* 14, no. 9 (2020): 2058‐2068, https://doi.org/10.1002/1878‐0261.12686.

The above article, published online on 07 April 2020 in Wiley Online Library (wileyonlinelibrary.com), has been retracted by agreement between the journal Editor‐in‐Chief, Kevin Ryan; FEBS Press; and John Wiley & Sons Ltd. The retraction has been agreed following concerns raised by a third party. An investigation identified that the control, KU711 and 17‐AAG liver and kidney panels shown in Figure 6B were duplicated from another article published earlier by some of the same authors. The reused images were presented to represent a different cell line. The authors cooperated with the investigation and explained that this was an inadvertent error; they also provided some supporting data. However, the data provided was not sufficient to restore the editors’ confidence in the article's results and conclusions. Therefore, the editors consider the results and conclusions to be unreliable. The authors disagree with the retraction.

**RETRACTION**: C. Subramanian, P. T. Grogan, T. Wang, J. Bazzill, A. Zuo, P. T. White, A. Kalidindi, D. Kuszynski, G. Wang, B. S. J. Blagg and M. S. Cohen, “Novel C‐Terminal Heat Shock Protein 90 Inhibitors Target Breast Cancer Stem Cells and Block Migration, Self‐Renewal, and Epithelial–Mesenchymal Transition,” *Molecular Oncology* 14, no. 9 (2020): 2058‐2068, https://doi.org/10.1002/1878‐0261.12686.

The above article, published online on 07 April 2020 in Wiley Online Library (wileyonlinelibrary.com), has been retracted by agreement between the journal Editor‐in‐Chief, Kevin Ryan; FEBS Press; and John Wiley & Sons Ltd. The retraction has been agreed following concerns raised by a third party. An investigation identified that the control, KU711 and 17‐AAG liver and kidney panels shown in Figure 6B were duplicated from another article published earlier by some of the same authors. The reused images were presented to represent a different cell line. The authors cooperated with the investigation and explained that this was an inadvertent error; they also provided some supporting data. However, the data provided was not sufficient to restore the editors’ confidence in the article's results and conclusions. Therefore, the editors consider the results and conclusions to be unreliable. The authors disagree with the retraction.

